# Institutionalizing Digital Parenting Programs in Low Resource Settings in China: Comparative Case Study of Health Care and Education Sectors Using the RE-AIM Framework

**DOI:** 10.2196/79848

**Published:** 2026-01-06

**Authors:** Xinyu Shi, Ruochen Ruan, Yi Qie, Jamie M Lachman, Na Zhong, Zuyi Fang

**Affiliations:** 1 School of Government, Beijing Normal University, Beijing, China; 2 Faculty of Education, Beijing Norma University, Beijing, China; 3 China Development Research Foundation (CDRF), Child Development Research Institute, Beijing, China; 4 Department of Social Policy and Intervention, University of Oxford, London, United Kingdom; 5 Centre for Social Science Research, University of Cape Town, Rondebosch, Cape Town, South Africa; 6 Parenting for Lifelong Health, Barnett House, Oxford, United Kingdom; 7 Chengbei Preschool, Xinyu, China; 8 Institute of Population Research, Peking University, No.5 Yiheyuan Road, Haidian District, Beijing, 100871, China, 86 13716165860, 86 13716165860; 9 Department of Social Policy and Intervention, University of Oxford, Oxford, United Kingdom

**Keywords:** digital delivery, parenting program, implementation science, Reach, Effectiveness, Adoption, Implementation, and Maintenance, RE-AIM, child development

## Abstract

**Background:**

Digital parenting programs offer a promising way to disseminate evidence-based parenting knowledge and support early childhood development. They help reduce costs while improving scalability and fidelity. However, their successful implementation is context-dependent, and existing research offers limited guidance on how the implementation of digital parenting interventions unfolds across diverse settings.

**Objective:**

This study aims to identify the shared and unique facilitators and barriers affecting each dimension of implementation, as well as differentiated mechanisms that support the effective implementation and institutionalization of such interventions across diverse settings.

**Methods:**

Using a multiple-case study design, this research compared the implementation of a digital (chatbot-led) parenting program across 2 distinct settings in China: urban educational and rural health care contexts. The intervention content remained consistent, while the contexts and formats of local human-led support differed. Guided by the RE-AIM framework, this study examines the program’s reach, adoption, implementation, and maintenance in both settings. Data sources included program documents, field observations, semistructured interviews, and focus group discussions with 83 stakeholders. Thematic analysis was conducted using ATLAS.ti until thematic saturation was reached.

**Results:**

Data were collected from 83 stakeholders, and findings are based on an analysis of 18 interviews and 4 focus groups with caregivers, village doctors, and health officials from the rural health care setting, and 29 interviews and 4 focus groups with caregivers, teachers, social workers, and managers from the urban educational setting. Regarding reach, strong relationships between parents and implementers and the credibility of program developers were shared facilitators in both settings. Parenting conservatism and limited understanding of the program were shared barriers. In rural health care settings, parents’ perception of village doctors as lacking parenting expertise posed an additional challenge. For adoption, trust between managers and program developers, program alignment with organizational functions, and organizational empowerment supported implementation are shared facilitators in both settings. At the individual level, task-driven motivation helped, while time constraints hindered adoption in the health care setting. Teachers adopted the program due to its relevance to their roles in the educational setting, unlike village doctors, who did not see it as part of their core duties. For implementation, supportive management and clear guidelines were shared facilitators in both settings, while a lack of purpose and psychological pressure acted as barriers. Rural implementation was aided by scheduling during off-seasons and standardized workflows, whereas flexible workflows were essential in the educational setting. Regarding maintenance, alignment with organizational functions and internal resources facilitated sustainability in both settings, while overreliance on government authorization posed challenges. Educational settings required contextual adaptation, while health care settings needed more content adaptation.

**Conclusions:**

Implementing digital parenting programs is a complex process, influenced by multilevel facilitators and barriers that vary across regions (rural vs urban) and settings (educational vs health care). This study highlights the importance of context-specific implementation strategies and proposes differentiated delivery models tailored to local structures and needs.

## Introduction

Early childhood is a critical period for long-term educational, emotional, and economic development [1]. Globally, more than 43% of children younger than 5 years of age are at risk of not reaching their full developmental potential, with children in low- and middle-income countries (LMICs) being disproportionately affected [2]. The failure to achieve developmental potential during early childhood has far-reaching consequences, not only for individual well-being but also for societal progress, which leads to losses in human capital and increased risks of social instability [3]. These challenges underscore the critical importance of investing in effective strategies that support ECD.

Among the most influential predictors of positive ECD outcomes is the quality of parenting [4,5]. Improving parenting practices, especially in under-resourced settings, is therefore a key priority in global ECD efforts. Parenting interventions, which are typically grounded in social learning and attachment theories, aim to equip parents with the knowledge and skills necessary to enhance parenting quality and to foster home environments that promote children’s health and development [6-8]. Such programs have been shown to improve ECD outcomes, enhance positive parenting practices, reduce the use of harsh or violent discipline, and improve caregivers’ mental health [9,10]. Meta-analytic findings indicate that such interventions in LMICs yield greater improvements in children’s cognitive, language, and motor development, highlighting the potential for large-scale benefits in resource-limited settings [10].

Traditional parenting interventions, delivered through home visits or small-group sessions, are resource-intensive and face barriers such as time constraints, geographic distance, and limited personnel [3,11]. These limitations make conventional delivery methods difficult to scale and sustain, particularly in low-resource settings [12,13]. Nowadays, the growing accessibility of the internet and the widespread ownership of mobile phones present a promising opportunity to overcome these challenges by leveraging digital technology [14]. Digital parenting programs provide a cost-effective way to deliver evidence-based parenting support, particularly in low-resource settings, by enhancing scalability while maintaining high fidelity [12,15]. They reduce costs associated with time and travel and increase accessibility to high-quality parenting resources [16,17]. Emerging evidence indicates that digital parenting interventions can positively influence multiple outcomes, including child development, parental confidence, parenting stress, and children’s behavioral problems [18-20]. These programs are particularly effective when complemented by human-led support, either in person or remotely [21].

The success of social and behavioral interventions hinges not only on the inclusion of key components designed to facilitate behavior change, but also on the fidelity and quality of their implementation in real-world settings [22]. While the body of research demonstrating the effectiveness of digital parenting interventions in promoting child development is growing, limited attention has been given to their implementation processes. This gap in knowledge poses significant barriers to the replication, adaptation, and scale-up of such interventions, particularly in diverse and resource-constrained contexts [23,24].

Implementation research seeks to uncover what works, how it works, and why it works in everyday settings beyond the confines of controlled trials [25]. It emphasizes identifying contextual factors that influence successful implementation, such as characteristics of the delivery environment, community context, interorganizational partnerships, and intervention delivery mechanisms [26,27]. The RE-AIM (Reach, Effectiveness, Adoption, Implementation, and Maintenance) framework is a widely recognized model that offers a comprehensive approach for evaluating the implementation and public health impact of interventions [28]. It helps researchers and practitioners identify the strengths and barriers in intervention implementation, thereby facilitating the optimization and broader dissemination of public health practices [28-30]. This approach also offers insights into how short-term programs can be transformed into sustainable, institutionalized practices within organizations and service systems [31]. While existing studies applying the RE-AIM framework have examined digital interventions in various health domains, such as diabetes management, psychotherapy, vaccination, and health behavior promotion [32-35], there is a notable gap in research on the implementation of digital parenting programs, especially across different settings. This gap is especially evident in LMICs, where such interventions remain underexplored and underdeveloped.

Guided by the RE-AIM framework, this study addresses these gaps by evaluating the implementation of a digital parenting program, “Keyushiguang”, in 2 distinct service delivery systems in China: an urban educational setting and a rural health care setting. In China, where significant early childhood development gaps and resource disparities persist, there is an urgent need for scalable, context-sensitive parenting support models [36,37]. This study, therefore, examines the program’s reach, adoption, implementation, and maintenance to identify shared and setting-specific facilitators and barriers. By comparing these 2 delivery models, the study aims to provide new insights into the differentiated mechanisms required for scaling and sustaining digital parenting support in diverse, low-resource contexts. The program’s impact evaluation will be reported separately.

## Methods

### Study Design and Context

A multiple-case study approach was used to facilitate a deeper understanding of individual cases while also identifying overarching patterns. A case study is an empirical research method used to investigate contemporary phenomena within their real-world context. By analyzing a specific case in depth, researchers gain insights into the background, processes, and influencing factors, which is especially valuable for studying complex social phenomena or contexts [38]. Case studies can be categorized into single-case and multiple-case studies, depending on the number of cases analyzed. The multiple-case study approach was adopted to enhance the validity of findings from different cases, leading to more robust and reliable conclusions [39]. This method allowed us to identify commonalities and differences in the facilitators and barriers to implementation across different contexts.

### Theoretical Underpinnings: RE-AIM

This paper focuses on 4 key dimensions of the RE-AIM framework: Reach, Adoption, Implementation, and Maintenance. Effectiveness was assessed using quantitative methods and will be presented separately.

Reach refers to the total number, proportion, and representativeness of individuals who are willing to participate in a given initiative, intervention, or program [28]. It primarily focuses on understanding the factors that influence individuals’ decisions to engage—or not engage—with the program. Adoption refers to the absolute number, proportion, and representativeness of settings and intervention agents (people who deliver the program) who are willing to initiate a program, as well as the reasons behind their decisions [28]. Implementation primarily focused on the program’s fidelity, which is the extent to which the program is delivered as intended, and examined how this fidelity was achieved throughout the delivery process [40]. Maintenance at the setting level of a program refers to its institutionalization or integration into routine practices and policies [30].

This paper evaluated each of these dimensions across the 2 distinct settings, analyzing the factors that were perceived to facilitate or hinder participation in the programs, as well as the differences in these factors between the 2 settings.

We also reported a descriptive overview of reach, adoption, and implementation. Reach was calculated by the ratio of participants who consented to participate relative to the total number of eligible individuals in both settings. The recruitment criteria for both programs were similar, with the primary requirement being that the caregivers of children be the primary caregivers. Adoption at the staff level is calculated by dividing the number of staff who agreed to participate in program implementation by the total number of staff eligible to participate in the program within the organization. In addition, we assessed the implementation based on the fidelity checklists of implementers.

### Case Selection

The selection of cases in this research was guided by the study objectives. This paper examines the implementation of the digital (chatbot-led) parenting intervention in 2 distinct settings in China: an urban preschool (educational) setting and 2 rural health centers (health care) settings. Intervention delivery in both settings was led by local implementation partners, with support from the research team that developed the digital intervention. Consequently, the intervention’s digital delivery method and content remained highly consistent across the 2 settings, with only minor adaptations made for the rural health care setting, such as colloquial expressions used, without altering the core parenting principles.

Case boundaries were defined as follows. The urban educational case referred to the implementation within one preschool in Xinyu city, Jiangxi Province, while the rural health care case referred to the implementation within the village-level public health system, supported by the county-level Maternal and Child Health Department and 2 township-level Health Centers in Huining County, Gansu Province. The analysis covered the full pilot implementation period: the preschool intervention that ran from March 2024 to June 2024, and the health care intervention that ran from October 2024 to January 2025. Stakeholders included in the case analysis were those directly engaged in program implementation—caregivers, teachers, social workers, village doctors, program managers, and local government officers. Broader community members who were not directly involved in program delivery were excluded.

The digital parenting program “Keyushiguang” was culturally adapted from the Parenting for Lifelong Health’s ParentText chatbot for parents of children aged 2 to 9 years by a local research team [41]. Delivered via a rule-based chatbot on WeChat, the program covers 8 key parenting topics, each consisting of 3 to 6 modules depending on the child’s age. Each module includes an introduction, quizzes, core parenting tips (in video or text format), and home exercises. The chatbot sends one parenting module every 23.5 hours, allowing parents to gradually develop their parenting skills and apply them in daily situations. Designed to integrate smoothly into parents’ daily routines, the chatbot enables flexible engagement at convenient times, such as after work, during breaks, or before bedtime. This gradual approach encourages parents to apply the learned skills in real-life scenarios, such as managing child behavior or establishing daily routines. By offering relevant, practical, and easily accessible support, the chatbot helps parents stay engaged consistently, enhancing the program’s long-term impact (Detailed program description can be seen in Multimedia Appendix 1).

The main distinctions between the 2 implementations lay in the format of additional human-led parenting support and were driven by the unique implementation contexts and available resources in each setting. In the urban educational setting, chatbot-led digital delivery was supplemented by message-based WeChat group interactions via web, held once or twice per week, and facilitated by headteachers and trained social workers. In contrast, the rural health care setting incorporated biweekly 40-minute individual home visits conducted by village doctors to reinforce the content delivered online and foster engagement.

The choice between group-based and individual-based human-led parenting support models was based on a comprehensive assessment of the target population and implementation environment [42]. In China, rural areas typically face greater challenges to child development than urban areas [43]. Therefore, the program was designed as a universal intervention (available to all families regardless of child development status) in the urban setting, while in rural areas, it was implemented as a selective intervention (targeted at high-risk families). Additionally, resource feasibility and cultural acceptance also informed program design. For example, most rural families have access to village doctors, whereas urban preschools face teacher shortages. In addition, rural parents are more receptive to home visits [44]. Therefore, we adopted a group-based WeChat interaction model for the urban preschool setting and a combined individual and group home visit model for the rural health care setting to better suit local needs and contexts. The comparison of the 2 pilot implementations is provided in Multimedia Appendix 2.

The rationale for selecting these 2 cases was that by controlling for similarities in program content, the study could minimize the impact of content-related differences and more precisely analyze the result variations arising from differing implementation contexts (ie, urban education vs rural health care settings).

### Data Collection

We used data triangulation to enhance the comprehensiveness and validity of our findings. Multiple sources of data were used, including program documentation (such as program manuals and materials), field observation notes (such as the behaviors of stakeholders in the process of program pre-preparation, implementation, and evaluation), semistructured individual interviews, and focus group discussions (FGDs). Our triangulation involved 2 levels. First, we conducted within-case triangulation by integrating these diverse data sources to validate findings within each of the 2 settings. For example, we compared implementers’ subjective perceptions of their implementation fidelity with parents’ feedback (as reflected in the fidelity checklist) and triangulated these with our field observation records. This approach allowed us to more accurately interpret and evaluate the perspectives expressed by stakeholders. Following this, we conducted between-case triangulation (ie, a cross-case analysis). This second level systematically compared the validated themes from the urban educational setting against those from the rural health care setting to identify the overarching facilitators, barriers, and divergent patterns presented in our results.

Program-related documents retained since the program’s inception were collected and analyzed. These included program proposals, curriculum designs, daily implementation records, and training materials, which served as foundational resources for understanding the intervention. All documents were reviewed systematically, and key implementation details were extracted for analysis. In addition, as members of the research team, the authors were actively involved in and directly observed the implementation processes in both settings, providing them with in-depth insights into the contextual dynamics and operational realities of the program delivery.

Field observations related to stakeholders’ behaviors, program structure, and implementation processes. We observed the behavior and actions of stakeholders throughout the program and recorded those, which allowed for a more accurate understanding of the viewpoints they expressed.

**Figure 1.**
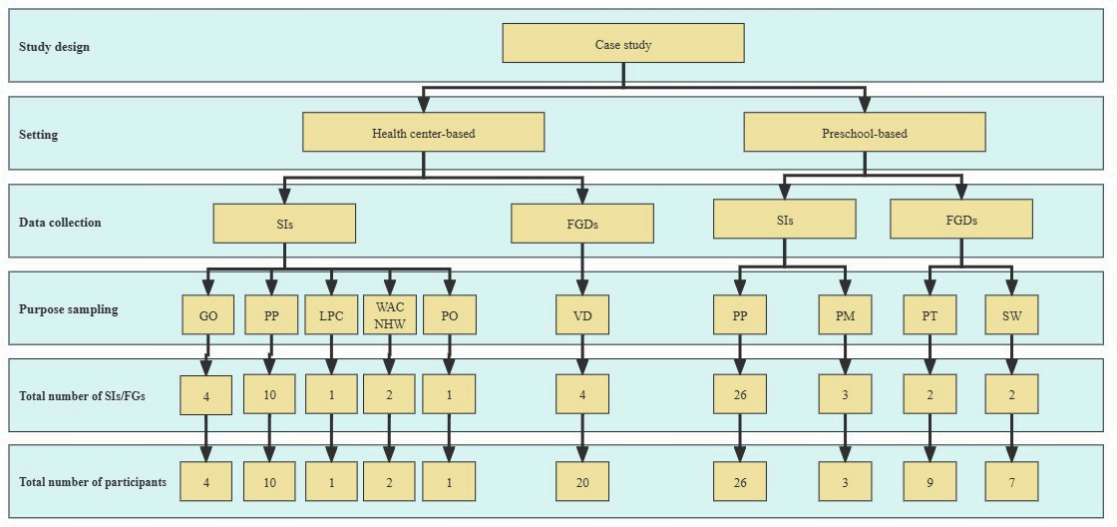
Stakeholder sampling and data collection design for the 2 cases.

We adopted a purposive sampling strategy, which is consistent with qualitative research practices that emphasize differentiation (Figure 1). Participants were deliberately selected to reflect diversity in roles, experiences, and contextual backgrounds. This approach allowed us to explore the phenomenon under investigation in a more nuanced and comprehensive manner. Specifically, participants were categorized into 5 key stakeholder groups: program officers, local leadership, local program managers, program implementers, and program participants. The final sample included 1 program officer from the health care setting; 7 local program leaderships (ie, 3 preschool leaderships and 4 government officers from the health care setting); 3 local program managers (ie, 2 Women’s and children’s health workers and 1 local program staff from the health care setting); 36 program implementers (ie, 9 preschool teachers and 7 social workers from the preschool setting, and 20 village doctors from the health care setting); and 36 program participants (ie, 26 caregivers from the preschool setting; and 10 caregivers from the health care setting).

The figure 1 illustrates the overall structure of data collection. As detailed in the Methods section, the implementation and data collection were conducted sequentially (urban case first, followed by the rural case). FGD: focus group discussion; GO: government officers from the healthcare setting; LPC: local program coordinator; PM: preschool managers; PO: program officer; PP: program participants; PT: preschool teachers; SI: semistructured interview; SW: social worker; VD: village doctor; WACHW: Women's and Children's Health Worker.The interview and FGD guides were developed based on the RE-AIM framework, incorporating a structured set of open-ended questions. Data related to the “Reach” dimension were primarily obtained from program participants, while “Implementation,” “Adoption,” and “Maintenance” were explored through interviews and focus groups with program donors, local leadership, local program managers, and program implementers. Each FGD lasted approximately one hour, and individual semistructured interviews averaged 40 minutes in duration. Prior to data collection, informed consent was obtained from all participants. With participants’ permission, all interviews and FGDs were audio-recorded. Recordings were subsequently transcribed, reviewed for accuracy, and anonymized to ensure confidentiality.

### Data Analysis

**Figure 2.**
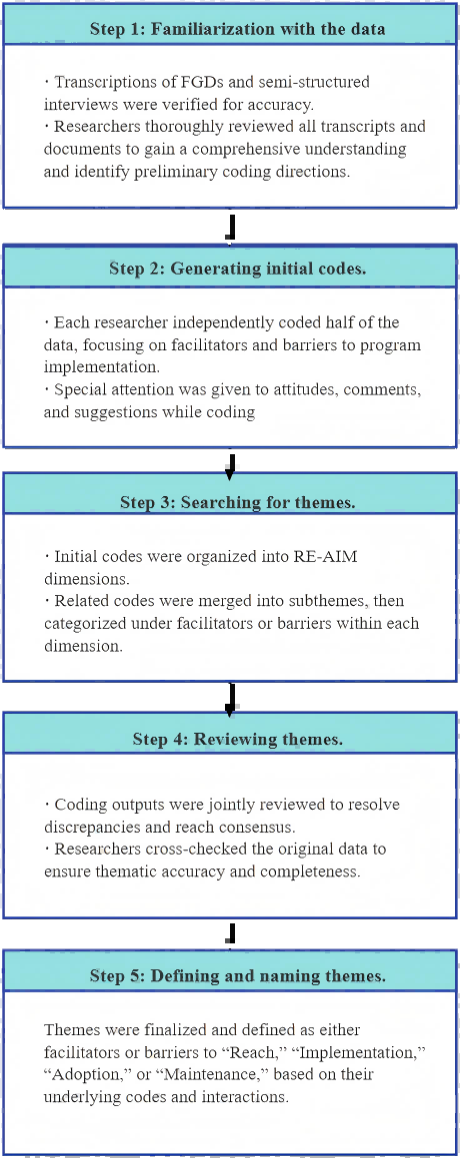
The process of data analysis.

Data were coded using ATLAS.ti (version 8; Lumivero). A thematic analysis was conducted using a hybrid inductive-deductive approach to identify patterns across the dataset [45]. As data collection was conducted at different times, we first collected and analyzed data from the urban educational setting following a stepwise thematic analysis procedure. After completing data collection in the rural health care setting, we applied the same analytical process. To ensure consistency and mitigate the risk of bias from this sequential design, the same coders applied the same predefined RE-AIM framework to both datasets independently. Only after both independent analyses were complete were the results from the 2 settings formally compared to identify similarities and differences. The coding process was conducted by 2 researchers (XS and RR) and followed the steps outlined by standard thematic analysis [45,46]. Thematic analysis was conducted through a structured 5-step process—data familiarization, initial coding, theme development, collaborative review, and final theme definition—guided by the RE-AIM framework to identify facilitators and barriers to program implementation (Figure 2). During the coding process, we continuously compared new data with existing codes and themes. We considered thematic saturation to be achieved when no new codes or themes emerged from subsequent interviews and FGDs. Given the diversity of stakeholders included—parents, teachers, village doctors, local managers, and government officers—the data provided a comprehensive reflection of perspectives across both settings.

Finally, as the capstone of our cross-case analysis, we synthesized the shared and setting-specific facilitators and barriers identified through the RE-AIM framework. This synthesis allowed us to construct the differentiated implementation models presented as the final component of our Results.

### Ethical Considerations

Ethics approvals were obtained from Beijing Normal University (approval numbers: SSDPP-HSC-2024003 and BNU202410100062) and the University of Oxford (SPI_DREC_24_006). We provided participants with a detailed introduction to the program and obtained their informed consent voluntarily. All data is stored in a secure database and is used only for research purposes by the researchers.

### Positionality Statement

We acknowledge that our dual roles as program designers and researchers may have influenced data interpretation. To mitigate this, we applied both personal and methodological reflexivity throughout the study.

Our research team has diverse academic backgrounds, including evidence-based social intervention, child development and protection, public administration, education, and social work. These perspectives shaped how we approached the research: team members with social intervention and public administration backgrounds tended to focus on macro-level structures and policies, while those with child development, education, and social work backgrounds were more attuned to participants’ lived experiences. To minimize potential biases from these disciplinary differences, the team engaged in ongoing reflexive discussions, considering multiple perspectives at all stages of analysis.

Methodologically, 2 researchers (XS and RR) independently coded different portions of the dataset and cross-checked each other’s work. A third researcher (ZF) reviewed the coding, provided feedback, and helped resolve discrepancies through iterative discussion until consensus was reached. This collaborative process ensured rigor and consistency in the analysis.

In addition, practical measures were taken to reduce researcher influence during data collection. Data collectors were trained to use semistructured interview guides with open-ended, nonleading questions, fostering a neutral environment that encouraged participants to share their authentic experiences.

## Results

### Overview

To ensure methodological rigor and transparency, the reporting of this study followed the iCHECK-DH: Guidelines and Checklist for the Reporting on Digital Health Implementations (Checklist 1) [47]. Descriptions of interview participants can be seen in Multimedia Appendix 3.

### Case Context and Stakeholder Profiles

#### The Urban Educational Setting: A Preschool in Xinyu City

This pilot was implemented in a preschool located in Xinyu City, an urban, medium-income area in eastern China. The preschool provides early childhood education before primary school, with parenting integrated into daily activities. The main participants were parents, most of whom hold stable jobs; their participation time was influenced by their work schedules. Key implementers included teachers who hold educational credibility and trained social workers who offered professional parenting support and assisted teachers in carrying out the program. The preschool leadership’s primary motivation was to enhance the school’s educational quality and reputation.

#### The Rural Health Care Setting: 2 Townships in Gansu Province

This pilot took place in health centers across 2 townships in Huining County, Gansu Province, a low-income rural region in western China. The centers are responsible for public health services, and promoting early childhood development was one of their key goals. The main participants were parents recruited from rural communities, most of whom engage in agricultural work. Their participation was shaped by the agricultural calendar and seasonal migration for work. The key implementers were village doctors who were trusted in their communities with strong local knowledge but generally older and less experienced in parenting. The health care leaderships were primarily motivated by fulfilling national public health directives. We labeled the interview data based on the source and role of the interviewees. The initial letters in each label represent the interviewee’s role, as illustrated in Figure 1. HB and SB denote data from the hospital-based program and preschool-based program, respectively. The last 3 digits of each label indicate the document number, and each number corresponds to a unique interviewee or focus group.

### Reach—Facilitators and Barriers

#### Overview

In total, 541 (81.4%) caregivers (402 [74.3%] were mothers, 135 [25%] were fathers, and 2 [0.3%] were grandparents) of the 665 eligible families consented to participate in both the program and the research.

In contrast, the township central health center–based program aimed to recruit 120 parents of children aged 24‐59 months from 2 pilot townships, out of approximately 795 eligible families. A total of 83 out of the targeted amount of 120 were able to participate (69.2%).

#### Shared Facilitators to Reach

The main motivations for participation in the program include its strong emphasis on child development, strong relationships between program implementers and caregivers, the perceived credibility of the program developers, and the convenience of learning through a chatbot.

A primary motivation in both settings was a recognized need for practical parenting skills. In the rural health care settings, caregivers expressed that they faced ongoing challenges in daily parenting and felt that their current knowledge was insufficient. They recognized the need to learn practical parenting skills to better support their children. In the urban educational setting, parents expressed similar concerns.

We don’t know much about how to support our children’s growth or deal with their emotional problems. This program gives us a chance to learn parenting ways.

[PP, HBD05]

Another shared facilitator was the influence of implementers, driven by social relationships. Some caregivers in the health care setting reported that their agreement to participate was influenced by their trust and social pressures from program implementers, such as face-saving norms, social obligations, or a sense of personal favor. Similarly, in the educational setting, the motivation to participate also stemmed from the influence of teachers.

The village doctor is our family doctor. He has helped us a lot and has a good relationship with us. He wants us to join the program, so we are willing to support him.

[PP, HBD11]

At first, the teacher encourages us to participate in this parenting program. I think if other children are participating, we shouldn’t seem uncooperative with the teacher’s efforts.

[PP, SBD11]

Parents in the health care setting also considered the background of the program developers. Knowing that the program was developed by a research team from a prestigious university in China enhanced its credibility and increased parents’ perceived benefits. Similarly, parents in the educational setting shared the same view, trusting the program’s credibility due to its association with a reputable institution, which further reinforced their belief in the program’s potential benefits.

There is too much parenting information online, and it is often hard to tell what is true or false. But this online course is developed by a professional team, so we feel the content is reliable and will be helpful to me.

[PP, HBD10]

The convenience of learning was also an essential factor for program reach. Parents in both educational and health care settings appreciated that the chatbot-based delivery allowed them to access the content anytime and anywhere. The use of diverse formats, such as videos and text, accommodated different learning environments and individual learning preferences.

The timing is fine because it is flexible. You can choose when to learn, so it is convenient when you have free time.

[PP, SBD15]

The course offers both video and text options, which is suitable for different scenarios. When I have free time, I choose to read the text. When I am busy, I play the video and listen to the content.

[PP, HBD11]

#### Shared Barriers to Reach

In both settings, shared factors hindering parents’ participation include parenting conservatism, a lack of understanding of the program, and limited time for learning.

A shared barrier in both settings was ‘parenting conservatism,’ where parents preferred to stick to traditional parenting methods. During the program promotion process, some parents in the educational setting with low engagement exhibited a form of ‘parenting conservatism’. They preferred to maintain their existing parenting practices and felt it was unnecessary to learn about other approaches, believing that new practices might not be suitable for their own situations. Similarly, in the health care setting, some caregivers also displayed resistance to adopting new parenting knowledge, preferring to rely on their established practices.

I feel that every family has its own parenting style. My other children are raised according to my own parenting methods, and there is no need to change it.

[PP, SBD13]

Furthermore, a limited understanding of the program served as a barrier in both contexts. Some caregivers in the educational setting indicated that they did not fully understand the program, such as its content, learning format, and flexibility. Some caregivers claimed they had no spare time to engage with the digital program and chose not to participate, unaware that the learning sessions were brief and could be easily completed anywhere in just a few minutes.

I think the course will take up a lot of time. Since I have to work and take care of my family, I don’t have much spare time for studying.

[PP, SBD13]

While some parents in the health care setting remained unaware of what they could gain from the program, this ultimately reduced their enthusiasm for participating.

At first, they don’t tell me the specific details of the program. I don’t know its benefits, so I initially see it as just a favor to village doctors.

[PP, HBD13]

#### Distinct Facilitators and Barriers to Reach

The urban educational setting presented several unique facilitators. These included urban parents’ recognition of the parenting values conveyed by the program and their acknowledgment of the implementers’ credibility and rationale in delivering parenting knowledge. These factors played a significant role in enhancing parental engagement in the educational setting. However, such facilitators were not observed in the health center-based program.

Some parents in the urban areas reported that the parenting concepts and attitudes conveyed through the program resonated with their own parenting beliefs. In contrast, parents in rural areas rarely expressed this kind of value-level recognition.

I agree with the parenting concepts in the course, and I am willing to learn it.

[PP, SBD08]

The perceived professional authority of the program implementers influenced caregivers’ decisions to participate. Parents viewed preschools as appropriate platforms for parenting support, and headteachers were seen as credible and persuasive figures in promoting parenting knowledge.

This preschool and its teachers are the best in the area, and I trust the quality of both the preschool and the teachers. I believe their actions will be beneficial to me.

[PP, SBD18]

In contrast, the rural setting faced a unique barrier related to the implementers’ professional role. In rural areas where village doctors delivered the parenting program for the first time, some doctors noted that because their routine work is not typically associated with parenting, parents were skeptical about their true intentions behind offering such services.

The parents doubt why I invite them to join the parenting program. They ask if I have received any benefits from the program organizers.

[VD, HBD25]

The summary of barriers and facilitators to reach can be seen in Multimedia Appendix 4.

### Adoption—Facilitators and Barriers

#### Overview

At the setting level, since the program was conducted as a pilot, 1 urban preschool and 2 rural health centers agreed to participate upon invitation. At the staff level, in the preschool-based program, the primary implementers included headteachers and recruited social workers, all of whom joined the program in response to directives from their supervisors. In the health center-based program, the main implementers were 2 women’s and children’s health workers and 30 village doctors. Eventually, both maternal and child health workers participated, while 23 village doctors (76.7%) remained engaged, and 7 withdrew from the program. Overall, the adoption level of the program was relatively high.

#### Shared Facilitators for Adoption

At the setting level, relevant information was gathered through interviews with local leadership. The main shared factors that promoted adoption included trust in the program developer, alignment with organizational functions, and empowerment of the organization and staff.

A primary factor in facilitating successful cooperation and adoption in both settings was trust in the program developers. Leadership in the educational setting acknowledged that the developers’ affiliation with a prestigious university in China enhanced their perception of the program’s credibility and value. In the health care setting, management also demonstrated trust in the collaborating research team.

You are a research team from a top university in China, and the quality of what you developed is guaranteed. We also have confidence that parents will accept the program, and we believe it will have a positive impact.

[PM, SBD33]

Another shared facilitator was the importance of the alignment between the program and the implementing organization’s core functions. In the educational setting, leaders reported that before adopting the program, they would assess whether their organization possessed the capacity and resources to support implementing the program and whether the initiative aligned with their institutional mandate. For instance, preschools regarded parenting services as a fundamental aspect of their work, viewing it as consistent with their educational responsibilities.

This is something I want to do. In 2010, our preschool established an ‘Early Education Center,’ and parenting is a part of our work.

[PM, SBD32]

Likewise, in the rural setting, health centers considered parenting support closely related to child health care and broader public health objectives.

This online-based parenting program focuses on promoting health education, especially concerning children’s growth and development. This is also connected to our daily work in public health.

[GO, HBD32]

Local leadership in both settings also highlighted its potential to empower staff and foster organizational growth. In the educational setting, managers noted that the program could enhance headteachers’ professional skills in parenting, positioning it as a valuable resource for supporting parents.

Our teachers gain many benefits during the implementation of the program, such as learning about family education and strengthening their connection with parents.

[PM, SBD30]

In the health care setting, leaderships emphasized that the program could strengthen the capabilities of village doctors as well as maternal and women’s and children’s health workers in delivering comprehensive children’s healthcare services.

Our county pays great attention to the implementation of early childhood development work in rural areas. Involving village doctors and other staff in this parenting program allows them to gain experience in carrying out such work.

[GO, HBD27]

At the staff level, both task-driven (motivation that arises from external obligations to complete tasks assigned by others, such as supervisors or program coordinators) and intrinsic motivation (motivation from personal interest or intrinsic desire) were key shared factors promoting adoption. For example, in the health care settings, implementers reported that the program was perceived as a mandated task from higher-level leadership, who required them to carry out the program and follow the specified timeline and procedures.

Our leaders attach great importance to this program and repeatedly emphasize the need to cooperate with the program developers to complete it. We feel that this is a very important task for us.

[VD, HBD24]

Intrinsic motivation also played an important role. Some implementers in the health care setting expressed that they recognized the significance of this pilot and the potential benefits it could bring to caregivers. This sense of mission motivated them to actively adopt and support the program. In the educational setting, teachers also felt that the program could support the development of both parents and children, contributing to better educational outcomes.

Another reason I participate in this program is that I feel what we are doing is meaningful. In our rural area, such resources are very precious, and many parents also have a demand for parenting knowledge. By completing this program, we can provide help to children and their families.

[VD, HBD31]

#### Shared Barriers to Adoption

A major shared factor hindering adoption in both settings was the perceived difficulty of implementation due to limited time. Both implementers and local leadership expressed concerns that implementers were already occupied with their regular responsibilities, which consumed most of their time. The entire program’s especially tight schedule further compounded the issue, as they were expected to complete tasks within a short timeframe. This increased the pressure on implementers, making them feel even more stressed.

Our village doctors have a lot of work and are very busy. We have to both prescribe medical treatment and complete public health tasks such as chronic disease management and vaccination. The program requires us to complete the tasks within a short time, and it feels very stressful.

[VD, HBD25]

We also have a lot of teaching tasks at school to complete, and the time is not very sufficient.

[PT, SBD34]

#### Distinct Facilitators and Barriers to Adoption

At the staff and setting level, prior successful collaboration experiences served as a unique facilitator for the health centers’ adoption of the program. However, health center-based implementers often lacked familiarity with parenting knowledge, and some faced difficulties in recruiting appropriate participants. The perceived difficulty in recruitment hindered program adoption among village doctors. Moreover, remuneration emerged as a significant motivating factor for implementers in the health care setting. In the educational setting, the teachers’ professional roles in parenting acted as a key facilitator.

At the institution level, factors promoting adoption differed between the 2 contexts. In the health center-based setting, health care leaders and program officers highlighted that prior successful collaboration experience played a key role. The local government, supported by the donor, had previously implemented similar ECD parenting programs in other townships within the county, which helped establish mutual trust and efficient working mechanisms.

They agree because we have previously implemented an early childhood development program here. We are familiar with the local leaders and institutions, and have built a strong trust relationship.

[PD, HBD35]

At the staff level, the relevance of the task to the implementers’ professional roles directly influenced their motivation to adopt the program. In the urban preschool setting, headteachers reported that parenting support was closely tied to their core duties, contributing to child development and the enhancement of educational quality. This strong connection fostered greater intrinsic motivation among them.

We also provide some parenting education in daily work. I review this program and feel it is quite good. If parents attend, it will benefit them.

[PT, SBD34]

In contrast, some village doctors and maternal and child health workers in the health care setting felt their routine work was less directly related to parenting. Consequently, they viewed the program more as a task assigned by higher-level leadership, with task-driven motivation playing a prominent role.

We have not done similar work before. I believe it is not our duty, as we are mainly responsible for public health. The main reason for implementing this program is that it is assigned by our leadership.

[VD, HBD24]

Some village doctors also reported difficulty in identifying parents who met the program’s participation criteria and were able to join. This challenge led to frustration and, over time, caused some of them to gradually withdraw from participating in the program altogether.

The main challenge for me is that I really cannot find suitable parents, because many children in the village are migrant or left-behind children.

[VD, HBD30]

Additionally, village doctors noted that the remuneration provided by the program officers was an important incentive for them to complete their responsibilities.

We carry out the task as required and receive some compensation. If it is voluntary, I will not be willing to complete the task.

[VD, HBD24]

The summary of barriers and facilitators to adoption can be seen in Multimedia Appendix 5.

### Implementation—Facilitators and Barriers

#### Overview

The investigation of implementation primarily focused on the program’s fidelity, which is the extent to which the program is delivered as intended, and examined how this fidelity was achieved throughout the delivery process [40].

The program was delivered with high fidelity in both settings, as demonstrated by fidelity checklists. In the preschool setting, headteachers recruited parents and reminded them to complete the online modules, while 10 social workers facilitated group discussions and completed all assigned tasks. In the health care setting, village doctors conducted home visits, supported by maternal and child health workers. Follow-up phone calls with 74 parents confirmed the content of the visits. The village doctors achieved an average fidelity score of 7.81 out of 8, indicating strong adherence to the program protocol.

#### Shared Facilitators for Implementation

Several key shared factors were identified as promoting the fidelity of program implementation: adequate onboarding training, a supportive management system, timely external support, and clear work guidelines.

Adequate onboarding training was a shared facilitator in both settings. Implementers in the health care setting believed that the training helped them understand the parenting principles, tasks, potential challenges, and proposed solutions. They also noted that the time allocated for discussion created opportunities to express concerns, ask questions, and receive prompt responses. Similarly, in the educational setting, teachers and social workers affirmed that the training enabled them to understand their respective tasks and effectively contribute to the program’s implementation.

The training before the program starts is essential. It helps us understand what we need to do, and the subsequent discussion sessions also address some issues.

[VD, HBD24]

A supportive management system involved a balanced combination of appropriate incentives and supervision to facilitate the successful implementation of the intervention. In both health care and educational settings, local leadership perceived that, to enhance fidelity, implementers required suitable incentives that align with their expectations, including both material and intrinsic (spiritual) rewards.

After all, the main work of village doctors is not parenting. They have limited time and energy, so they need to be compensated.

[GO, HBD27]

Our teachers need to have a sense of achievement when doing this program. Even giving them a certificate is an effective encouragement for them.

[PM, SBD32]

Meanwhile, while supervision was crucial in both contexts, its focus differed. Local program managers in the health care setting emphasized that supervision was essential to achieve a balance between quality control and task implementation efficiency. They believed that supervision should cause necessary work pressure while allowing sufficient flexibility in how tasks are carried out. In the educational setting, the focus of supervision was more on ensuring timely task completion, with managers highlighting the importance of regularly reminding teachers to meet deadlines.

Village doctors work hard. Sometimes, tasks cannot be completed due to external reasons. When supervising their work, we have to both consider the quality and avoid discouraging their motivation.

[WACNHW, HBD26]

Providing timely external support to implementers was perceived to be a critical shared factor for successful implementation, which involves access to expert advice, consultations, and problem-solving assistance from external consultants or organizations when issues arise during program implementation. Implementers in the health care setting reported that timely and adequate support—whether material, psychological, or resource-based—from organizations and program developers helped alleviate their stress, overcome challenges, and enabled them to successfully complete their tasks. Likewise, in the educational setting, implementers affirmed that support from program developers and managers was crucial for the smooth execution of their work.

I feel that the support is quite sufficient. You are always there whether we or the village doctors have any questions. I feel touched.

[WACNHW, HBD26]

Finally, having clear work guidelines was a key shared facilitating factor. Implementers in both educational and preschool settings noted that well-defined procedures contributed to smoother implementation by clearly outlining what needed to be done, when, and how. This clarity made it easier for them to carry out their tasks efficiently.

The clear work guidelines let me know what I need to do, making it easier to carry out my work.

[VD, HBD31]

This program provides clear instructions, and the guidance in all aspects is very detailed. So, we can implement it well according to the guidance.

[SW, SBD27]

#### Shared Barriers to Implementation

A lack of sense of purpose and psychological pressure was perceived as key shared challenges faced by some implementers in both settings. Psychological pressures refer to stressors such as heavy workloads, tight deadlines, and the emotional burden of ensuring the success of the program. These pressures can negatively impact the quality of program delivery and the well-being of implementers. While leadership demonstrated a clear understanding of the program’s goals, some implementers lacked this clarity. During the implementation process, it was observed that certain implementers were unsure about the program’s objectives and the value it could provide to caregivers. This uncertainty at times led to self-doubt, which had a negative impact on their motivation. Further communication with implementers confirmed this observation—several social workers from the educational setting and village doctors from the health care setting expressed confusion about the purpose of their roles and the potential benefits their efforts could bring to families.

When conducting home visits, we do not know our purpose. What exactly are we trying to do?

[VD, HBD24]

You do not know your role in this process, but you must do it. Moreover, at this point, you can feel quite confused.

[SW, SBD28]

Implementers in both settings shared the challenge of generating demand and encouraging active participation in the program, which caused psychological pressure among implementers.

Because not everyone is enthusiastic about participating, I think the appeal of the content to parents is crucial. Is the content we are providing useful to parents? Is it something they want?

[SW, SBD27]

#### Distinct Facilitators and Barriers to Implementation

A unique facilitator in the health care setting was the timing of implementation, which aligned well with the villagers’ work schedules. However, in China, village doctors typically only conduct home visits for specific health care tasks. Also, in the health care setting, parenting home visits are irregular, and the long distances they needed to travel posed a challenge during program delivery. Furthermore, while clarity in work procedures was a shared facilitating factor across both settings, there was a notable difference in the need for implementation flexibility across the 2 settings.

First, in the rural setting, village doctors shared that the program was implemented during the off-season for farming, just before the Chinese New Year, a period when there was less agricultural work and many parents who normally worked away from home had returned. This created free time for caregivers to participate in the program.

Parents are generally at home in winter. As the weather is cold, there is no farming to do. Besides, they do not go out for labor during this season, so most of them stay home.

[VD, SBD24]

Second, village doctors also reported that the physical distance between households and their base locations created challenges in conducting home visits on time.

It is still too far, the road is inconvenient, and it is not easy to return.

[VD, D30]

Third, standardized workflow served as a double-edged sword, with its impact varying across settings. In the educational setting, especially among headteachers, implementers preferred greater flexibility. They expressed a desire to customize token economy systems to encourage daily participation in their own class and requested more adaptable web-based group interaction structures to better address the diverse needs of caregivers.

I feel that our online group discussions are a bit stiff. The content shared is fixed and does not capture parents’ interest. If we integrate additional parenting support with our daily activities, it will be better.

[PT, SBD35]

In contrast, implementers in the rural health care setting, particularly village doctors who were typically older and had limited experience with parenting support, preferred a highly standardized process. They valued having clear guidance on what tasks to perform, when to perform them, and how to carry them out.

The workflow you provided is very clear. It outlines each step, and we just need to follow it to complete the task.

[WACNHW, HBD26]

The summary of barriers and facilitators to implementation can be seen in Multimedia Appendix 6.

### Maintenance at the Setting Level—Facilitators and Barriers

#### Overview

This article primarily explores the factors that may facilitate or hinder the integration of the program into the established service system. In both settings, the leadership actively worked towards promoting the program’s integration into the existing service infrastructure.

#### Shared Facilitators to Maintenance at a Setting Level

The shared factors were the integration of the program into an organization’s daily operations, alignment between the program content and the organization’s core functions, the availability of sufficient and appropriate internal human resources, and the low cost associated with digital delivery.

First, alignment with the organization’s core mission was a key shared facilitator. Local leadership in the health care setting emphasized that the program’s alignment with their existing responsibilities was essential for successful institutionalization beyond initial adoption. By supporting the organization’s core mission, the program minimized additional costs and streamlined integration into routine work. Likewise, in the educational setting, managers emphasized that the parenting program was a natural fit for their institution’s existing work and goals.

Normalizing it means integrating it with our public health services, which does not create any work pressure for us or add any burden. It should also benefit our daily work.

[GO, HBD27]

Second, leadership in both settings believed they had access to a well-suited workforce for program delivery. Village doctors in the rural setting and headteachers in the urban setting were described as stable, professionally trained in child development, and capable of ongoing learning. Their regular contact with families and their established trust and authority within the community made them particularly suitable for engaging caregivers and carrying out the program.

The village doctor team can handle this program. On one hand, they have good relationships with the villagers. On the other hand, they possess a lot of medical knowledge and have the ability to learn.

[GO, HBD32]

Our teachers are well-suited to take on this program. They already handle educational work and can easily use it as a tool in their daily teaching.

[PM, SBD33]

Third, local leadership in the educational setting emphasized that the low cost of digital delivery was a key factor facilitating program integration. By delivering core content via web through a WeChat-based chatbot, the program minimized the need for intensive offline operations. This digital approach not only helped maintain content quality but also significantly reduced the workload and operational costs for implementing organizations, making the program more sustainable and scalable within existing structures. Similarly, in the health care setting, managers highlighted that controllable costs were crucial for sustaining the program in rural areas.

I think the advantage of the online format is that, firstly, it can push information in real-time and is more flexible. At the same time, since we are in the information technology era, parents also use their phones often.

[PM, SBD27]

#### Shared Barriers to Maintenance at the Setting Level

Shared barriers to integrating the program into the organization’s daily operations included institutional dependence on higher government authorization, challenges in sustaining staff motivation, and difficulties in generating parental demand for parenting support.

A primary barrier in both settings was the institution’s reliance on higher-level government approval. In the rural context, health care leadership noted that integration into routine services would require authorization from senior government officials overseeing health affairs.

To integrate the program into our daily work, we need approval from the senior leaders in charge of this work at the higher level.

[GO, HBD33]

Similarly, in the urban context, preschool managers indicated that continued implementation in preschools would depend on approval and support from the education department.

To integrate the program into the preschool’s daily work, we need support from the local education bureau leaders. It would be best to also gain official support from your university.

[PM, SBD32]

In addition, leaders in both settings expressed concerns about sustaining staff motivation over the long term. While staff had fulfilled their responsibilities during the pilot phase, there was uncertainty about how to provide sufficient and appropriate incentives to support ongoing, routine implementation once the program became part of daily work. This concern was echoed in the educational setting, where managers also stressed the need for incentives to maintain teachers’ continued engagement.

If the work is unpaid and voluntary labor, as mentioned by the village doctor, it is a challenge for the village doctor.

[GO, HBD27]

Another significant barrier was the lack of perceived demand from parents. Government officers and organizational managers from both settings expressed concern that many parents did not recognize the value or necessity of parenting support. Without sufficient parental buy-in, they feared that efforts to integrate the program into routine services would result in low participation rates and limited impact.

After all, we are in the countryside. Some parents have a higher level of awareness and think that interacting with their children is meaningful. However, some parents feel it is unnecessary; if the children can play alone and do not cause trouble, they have done their job.

[WACNHW, HBD26]

Many parents, when it comes to parenting, understand but not to a great extent, which causes a disconnect. They feel they somewhat understand but not fully, and accepting some new concepts is difficult.

[PM, SBD30]

#### Distinct Facilitators and Barriers to Maintenance at the Setting Level

A unique facilitating factor in the health center-based delivery was the close relationship between village doctors and local families, which enhanced trust and communication. However, implementation in this setting also faced a unique challenge in securing resources for sustainable material incentives. Both the health care and educational settings shared the need for further localized contextual adaptation of the program. Nonetheless, the focus of these recommended adaptations differed between the 2 settings.

In the rural health care setting, leaderships perceived that parenting programs facilitated by village doctors were feasible for sustained implementation in rural health care settings. They believed that village doctors, who often had strong, long-standing relationships with families—sometimes dating back to prenatal care—were uniquely positioned to gain family acceptance of the program and successfully conduct home visits. In contrast, the preschool program relied on teachers leading group-based remote support and did not require the same depth of ongoing personal relationships with students’ families as village doctors had.

In our rural areas, village doctors are like family doctors, closely connected to the families. People know each other well, and home visits are easy. In the city, living in apartment buildings makes it less convenient, and people are more guarded.

[GO, HBD32]

In addition, the approach to sustaining staff motivation differed between the 2 settings. In the educational setting, preschool managers noted that parenting work is closely aligned with headteachers’ core educational duties. As a result, integrating the program into daily operations was more natural and did not significantly increase their workload. Therefore, spiritual incentives, such as recognition and a sense of professional fulfillment, were seen as important for promoting long-term motivation.

Parenting is already part of our teachers’ work, so it does not add extra burden. However, some motivation is still needed, such as certificates or awards.

[PM, SBD33]

In contrast, in the health care setting, leadership emphasized that village doctors primarily focused on public health responsibilities. Parenting-related tasks were viewed as additional duties, which increased their overall workload. In this context, material incentives were considered more effective, as they better compensated for the extra effort required for sustained engagement. By comparison, for preschool teachers, parenting was closely integrated with their daily work. The program did not add to their workload but rather complemented it, serving as an educational tool to support their parenting-related tasks.

Our village doctors’ main work is in public health. This task adds extra duties for them, so compensation is needed.

[GO, HBD28]

I don’t think this is a burden for the teachers, as they are already engaged in education. Parenting is also familiar to them. They can adapt the program content into teaching tools based on their needs.

[PM, SBD30]

Furthermore, adaptations to each specific implementation context were perceived as important. In the preschool, leaderships suggested that for the program to be effectively integrated into daily work, the digital learning format, duration, and additional parenting support needed to be further adapted to better align with teachers’ schedules and classroom activities. This type of adaptation primarily involved organizational alignment, ensuring the program fit seamlessly within existing school routines.

To become part of our daily work, the program needs to align with the kindergarten’s teaching needs and integrate with our activities. Teachers have to be the leaders.

[PM, SBD30]

In the health care setting, health care managers believed that for long-term implementation, the program needed to be adjusted to include special groups, such as left-behind children and children cared for by grandparents, who make up the majority in the local area, and also need parenting support. However, this time, the adaptation focused more on tailoring the program to parents who stayed at home to care for their children, as a result of careful consideration of available implementation resources and the exploratory nature of the pilot as an initial test of digital interventions in rural areas.

Like in our area, most young parents go out to work, leaving grandparents behind. Therefore, these families need more services.

[GO, HBD33]

The summary of barriers and facilitators to maintenance at the setting level can be seen in Multimedia Appendix 7.

### Differentiated Models of Digital Parenting Program Implementation in Two Sectors

**Figure 3.**
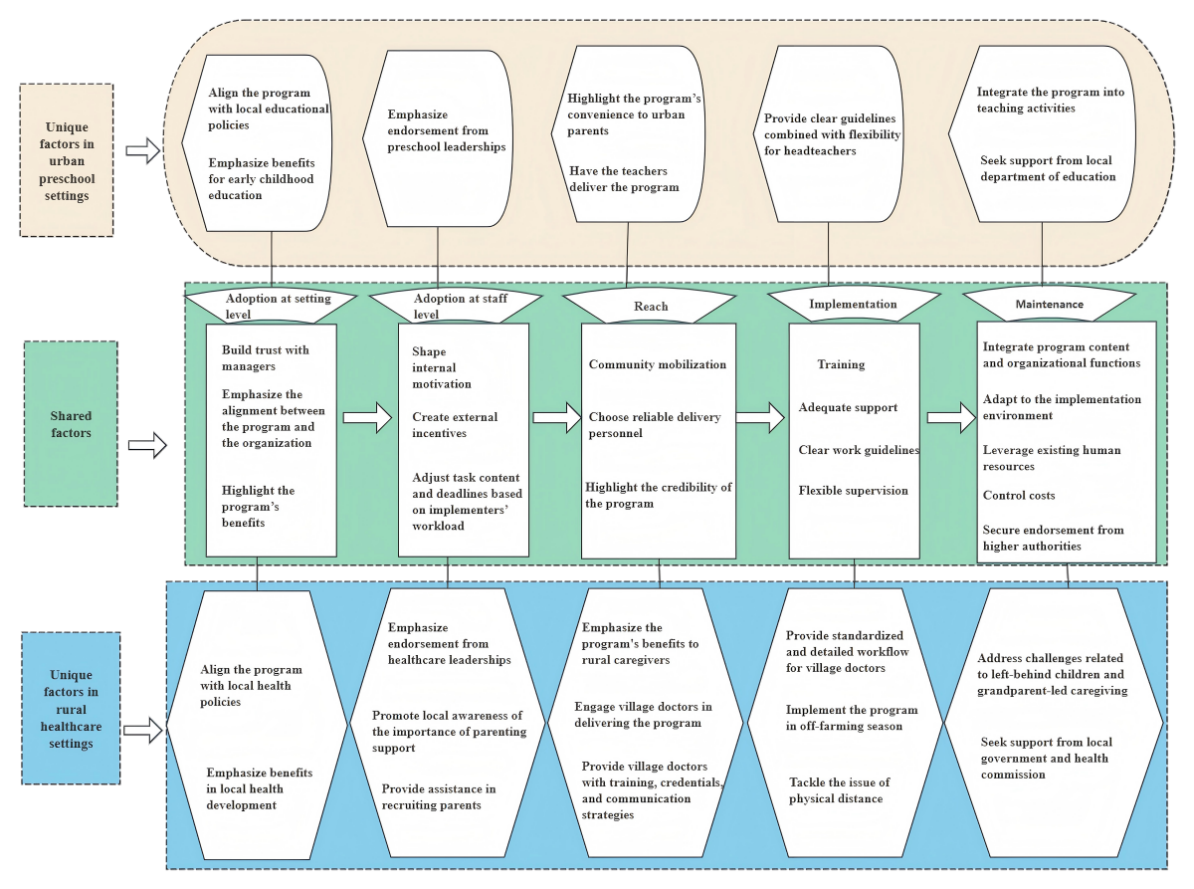
Differentiated models of delivery in urban preschool and rural health care settings.

Based on the results, we summarize two models for implementing and institutionalizing the digital parenting program in rural health care and urban educational settings (Figure 3). The basic sequence starts with promoting organizational adoption of the program, followed by encouraging staff adoption, then expanding reach. After that, the focus shifts to implementation and finally to maintenance. In impoverished rural areas, digital interventions need to be complemented by individualized human-led offline support to enhance the impact. Accordingly, we propose a model that involves collaboration with the health center and leverages village doctors to deliver the digital program alongside home visits. For urban areas, we suggest a model where digital components are combined with online parent groups led by teachers.

During the organizational adoption stage, building trust with managers, emphasizing the alignment between the program and the organization, and highlighting the program’s benefits are crucial. Trust can be fostered by demonstrating the evidence base and credibility of the program, which helps managers recognize the program’s potential. Emphasizing how the program aligns with the organization’s goals encourages adoption of the program and fosters positive expectations regarding its completion.

In rural health care settings, aligning the program with local health policies can strengthen its acceptance, particularly by emphasizing benefits such as improving early childhood development and addressing population health challenges. In contrast, urban educational settings can benefit from closer ties to educational policies, where the program’s role in enhancing teaching quality, promoting parent-teacher collaboration, and reducing behavioral problems in preschool environments is more prominent. Thus, the focus in rural areas is more health-centered, while urban settings prioritize educational outcomes.

At the staff-level adoption stage, it is essential to shape internal motivation, create external incentives, and adjust task content and deadlines based on implementers’ workload. First, effectively communicating the program’s significance helps foster internal motivation and enables implementers to recognize its benefits. Preschool teachers typically have stronger internal motivation because their daily work is directly related to parenting, allowing them to see immediate benefits. In contrast, village doctors in health centers, whose routine duties are less connected to parenting, require communication and training to understand how parenting relates to their role and to enhance their perception of the program’s importance.

Second, when internal motivation alone is insufficient, forming external incentives becomes essential. Depending on the program’s context, it is necessary to strike a balance between material and intrinsic incentives to fully motivate implementers. For example, in the rural pilot, providing village doctors with reasonable compensation effectively promoted their active participation in program implementation.

Regarding recruitment, urban preschools generally experience less difficulty, as parents tend to cooperate more readily with teachers. However, in rural areas, recruiting parents is more challenging due to complex family structures and village doctors’ limited experience with parenting work. This necessitates additional support from implementers and institutional leaders.

In the reach stage, community mobilization, choosing reliable delivery personnel, and highlighting the credibility of the program content are essential. Through community mobilization, parents can understand the program’s goals, content, and requirements, and most importantly, recognize the benefits of the program and that the learning process is convenient and low in time cost. In addition, it is worthwhile to highlight the program’s convenience to urban parents and emphasize its benefits to rural parents. During the promotion, it is also essential to highlight the program’s being systematic, distinguishing it from fragmented parenting information online. In urban preschools, teachers can serve as program implementers due to their close relationships with parents. In rural health care settings, village doctors can take on this role, as they are generally trusted by families. However, since parenting is not a routine part of village doctors’ responsibilities, providing them with adequate training, relevant credentials, and communication strategies is necessary to reduce parental skepticism about the program.

In the implementation stage, training, adequate support, clear work guidelines, and flexible supervision are essential for ensuring fidelity. However, the specificity of work guidelines should be tailored to the implementers’ roles. In rural health care settings, village doctors, who are generally unfamiliar with parenting work, require standardized and highly detailed workflows to guide their delivery. Conversely, urban preschool teachers, already experienced with parenting-related tasks, benefit from clear guidelines combined with flexibility to facilitate group discussions during parent meetings or school activities, supported by suggested discussion topics. Additionally, in rural areas, particular consideration must be given to the timing and logistics of home visits, as busy farming seasons often limit the availability of both village doctors and families for such visits.

In the maintenance stage, several factors are critical to ensuring the program’s sustainability: integrating program content with organizational functions, adapting to the implementation environment, leveraging existing human resources, controlling costs, and securing endorsement from higher authorities. Controlling costs is paramount, as high expenses threaten the long-term sustainability of the program.

Various strategies can be used to reduce implementation costs. Similar to adoption, alignment between organizational functions and program content is important, but in the maintenance stage, this alignment emphasizes better integration of the program with the organization’s daily work content and clarifying the position of the program task in the organization.

Using existing human resources by providing adequate onboarding and ongoing training can make the best use of the organization’s current staff rather than hiring new personnel. Furthermore, it is necessary to obtain endorsement from higher authorities, with preschools needing support from the education department and health centers from public health authorities.

Adaptations must reflect the distinct realities of urban educational and rural health care settings. In urban preschools, the program needs to be integrated into teaching activities, becoming a practical tool within educators’ daily routines. Conversely, rural areas must also address challenges related to left-behind children and grandparent-led caregiving to ensure these families are included in the services.

## Discussion

### Principal Findings

This study aimed to identify multilevel factors influencing the implementation of a digital parenting program across 2 distinct settings. The findings addressed this aim by revealing both shared and setting-specific facilitators and barriers across the RE-AIM dimensions, providing a comprehensive understanding of how digital parenting programs may be effectively implemented and sustained in different institutional contexts.

While well-known digital parenting programs such as Triple P Online and Incredible Years Online have shown positive outcomes in several countries [48-50], this evidence is predominantly from high-income countries, with limited exploration of their adaptability and sustainability across diverse implementation contexts. Our study directly addresses this gap by examining the implementation of a digital parenting program in 2 distinct settings in China—an urban educational setting and a rural health care setting—thereby contributing crucial evidence from a large low- and middle-income country.

Parents in both urban and rural areas expressed a desire for parenting knowledge, which was motivated by their wish to support their children in growing up healthy. Although the internet provided access to information, much of it was fragmented and lacked a systematic approach. As a result, parents often struggled to build a coherent knowledge system or apply these concepts in practice. This finding is consistent with previous research [51]. In contrast, the digital parenting program offers culturally adapted, evidence-based content that is systematic, practical, and easy to access.

Trust is indispensable to a program’s success, as previous study shows [52]. It must be built among organization managers, implementers, and the beneficiaries. First, building trust with managers and implementers to get their support is important [53]. It can be fostered by showcasing the program’s evidence base, emphasizing its benefits, and selecting those with prior cooperation experience. Second, building trust with beneficiaries requires the credibility of the delivery channel and the implementer. A trusted implementer can boost parents’ confidence in the program and provide extra motivations, such as social consideration (eg, face-saving or relationship building). In rural areas, village doctors, trusted as family doctors, are well-suited for this role [54], while in urban areas, preschool teachers hold similar credibility and respect.

Similar to previous research [55], we found that community mobilization for both parents and implementers before implementation is important for generating demand, thereby increasing the adoption and program reach. Parents should be introduced to the importance of evidence-based parenting, the benefits of the program, and how it works. At the same time, implementers need to understand the connection between the program and their routine work, its significance for them, and the necessity of the human-led components. Additionally, fostering a shared vision with all stakeholders is essential.

Providing support for implementers is also crucial, which aligns with previous research [56]. It is important to provide adequate training to equip them with implementation skills and clearly define their responsibilities. Establishing communication channels related to parenting support within the organization, such as regular meetings to discuss issues and digital systems to monitor progress. This will facilitate efficient vertical communication, supervision, and problem-solving mechanisms between implementers and organization managers.

Appropriate incentives for implementers are essential. Previous research emphasizes their importance, but it often lacks guidance on how to provide them [57]. We found that material and psychological incentives should be based on the workload and the fit between the implementer’s tasks and capabilities. When human-led responsibilities exceed usual duties, greater incentives may be required to ensure motivation and sustained engagement.

Building on these findings, several practical and theoretical implications emerge for strengthening digital parenting interventions across diverse contexts. Program adaptation is necessary for maintenance, which aligns with previous research [53]. Adaptation occurs at 2 levels: content and context [58]. Content adaptation involves adding information relevant to the organization’s functions and the local population’s needs, such as child nutrition and disease prevention in rural health care, and early education in urban preschools. Context adaptation involves adjusting delivery methods to fit the organization’s characteristics, ensuring that the program becomes integrated into regular work [56]. In rural areas, additional focus on left-behind children and grandparent caregivers is necessary because of their population structure [59]. While urban settings should incorporate the program into daily school activities, such as parent meetings and lectures.

### Implications for Policy, Practice, and Research

To better support families in both urban and rural areas, governments should invest in providing trustworthy and accessible parenting resources. Trusted institutions, such as preschools and health centers, can serve as effective channels for delivering this information. With government support and institutional credibility, families may be more likely to engage in the programs.

When implementing digital parenting programs, it is essential to tailor strategies to local contexts, considering organizational mandates, local culture, demographic profiles, and economic conditions. Integrating the program into existing institutional workflows can reduce costs and promote sustainability. Establishing trust with organizational leaders, frontline implementers, and the beneficiaries is also key and should be supported by early and continuous community mobilization and engagement. Importantly, implementation must strike a balance between fidelity to core content and flexibility in delivery. Aligning program delivery with the institutional capabilities and work patterns, while providing clear and structured workflows, can help maintain quality and adapt to implementers’ needs on the ground.

Future research should explore a broader range of settings and more diverse implementation strategies. Additionally, this study primarily used the RE-AIM framework to evaluate implementation at the mezzo- and macro-levels. However, parents, as direct beneficiaries, also play a crucial role. Their acceptance and participation significantly influence program success. Therefore, future research should adopt a micro-perspective focusing on parents’ perceptions, attitudes, acceptance, and engagement. Such insights would inform future implementation of digital parenting programs.

### Conclusions

This study’s findings go beyond demonstrating the feasibility of digital parenting programs in low-resource settings to highlight a key lesson for their institutionalization: successful scaling is not about a single “one-size-fits-all” digital solution. Effective implementation requires hybrid models that strategically combine low-cost technology with trusted local human infrastructure, such as teachers in urban schools or village doctors in rural clinics.

Our comparison of urban and rural settings shows that the human-led component must be carefully tailored. Urban environments can benefit from flexible online group support, whereas rural contexts often require the structure and accountability of in-person visits. These insights provide a roadmap for policymakers and practitioners to move beyond standardized rollouts and develop a flexible “implementation playbook.” By prioritizing adaptation to local social and organizational contexts, evidence-based digital parenting interventions can bridge the gap from efficacy to sustainable, equitable, and real-world impact.

### Limitations and Strengths

First, this study did not report on the program’s effectiveness. This may raise concerns about its impact. We plan to address this in a forthcoming publication specifically focusing on impact evaluation. Second, this study did not delve into micro-level aspects, especially parents’ adoption of and attitudes toward the program. While this is an important area, it could not be adequately covered due to space limitations and will be examined in future research. Third, this study was limited to 2 pilot sites. Including more case studies in future research will help enrich the findings and enhance external validity.

The strength of this study lies in its focus on the implementation process of digital parenting programs, providing valuable insights for future program implementation. We also explored different implementation models across urban and rural education and health care sectors. This can support potential scale-up in diverse contexts. Additionally, we discussed the use of human-led models in different practical settings. This can provide guidance for choosing appropriate approaches in future hybrid (digital and human-led) parenting programs.

## Supplementary material

10.2196/79848 Multimedia Appendix 1 Program description.

10.2196/79848 Multimedia Appendix 2 Comparison of the two programs.

10.2196/79848 Multimedia Appendix 3 Description of interview participants.

10.2196/79848 Multimedia Appendix 4 Summary of barriers and facilitators to reach.

10.2196/79848 Multimedia Appendix 5 Summary of barriers and facilitators to adoption.

10.2196/79848 Multimedia Appendix 6 Summary of barriers and facilitators to implementation of the digital parenting program.

10.2196/79848 Multimedia Appendix 7 Summary of barriers and facilitators to maintenance at the setting level of the digital parenting program.

10.2196/79848 Checklist 1 Checklist of iCHECK-DH guidelines. iCHECK-DH: Guidelines and Checklist for the Reporting on Digital Health Implementations.

## Abbreviations

FGD: focus group discussion
HBD: hospital-based program
LMIC: low- and middle-income country
PP: program participants
RE-AIM: Reach, Effectiveness, Adoption, Implementation, and Maintenance
SBD: preschool-based program
