# Supplementary material for: Institutionalizing Digital Parenting Programs in Low Resource Settings in China: Comparative Case Study of Health Care and Education Sectors Using the RE-AIM Framework
Source: J Med Internet Res. 2026 Jan 6;28:e79848. doi: 10.2196/79848 (PMC12772938; doi:10.2196/79848)

# Program Description

“Keyushiguang” is a parenting intervention program adapted from the Parenting for Lifelong Health (PLH) ParentText chatbot for parents of children ages 2 to 9 years. Program content is grounded in social learning and attachment theories. The cultural adaptation was conducted from June 2023 to March 2024 to ensure alignment with local family dynamics, the social environment, and parenting needs in China. The program covers eight parenting topics. Each topic includes three to six modules, for a total of 37 to 39 modules, depending on the child’s age. This content is delivered via a rule-based chatbot on WeChat. To strengthen the effect of the digital intervention, additional human-led parenting support was provided. In the urban educational setting, message-based WeChat group interactions were held once or twice every week; in the rural healthcare setting, trained village doctors conducted biweekly home visits, with each family receiving a total of five visits

The program covers eight core topics: parent-child relationships, child development, child behavior, learning, proactive parenting, child safety, family relationships, and parenting budgets. Each topic includes three to six modules, for a total of 37 to 39 modules, depending on the child’s age.

“Keyushiguang” is primarily delivered via a chatbot on WeChat, which sends parents one module every 23.5 hours to facilitate gradual skill-building. Each module consists of an introduction, quizzes, core parenting tips (presented in video or text format), and home exercises. Additional features include personalized troubleshooting, a developmental disability screening tool, a parent-child game library, localized resources and service contacts, and emergency support activated by specific keywords. The program lasts 2.5 months (see figure S1).

Figure S1. The process of digital intervention


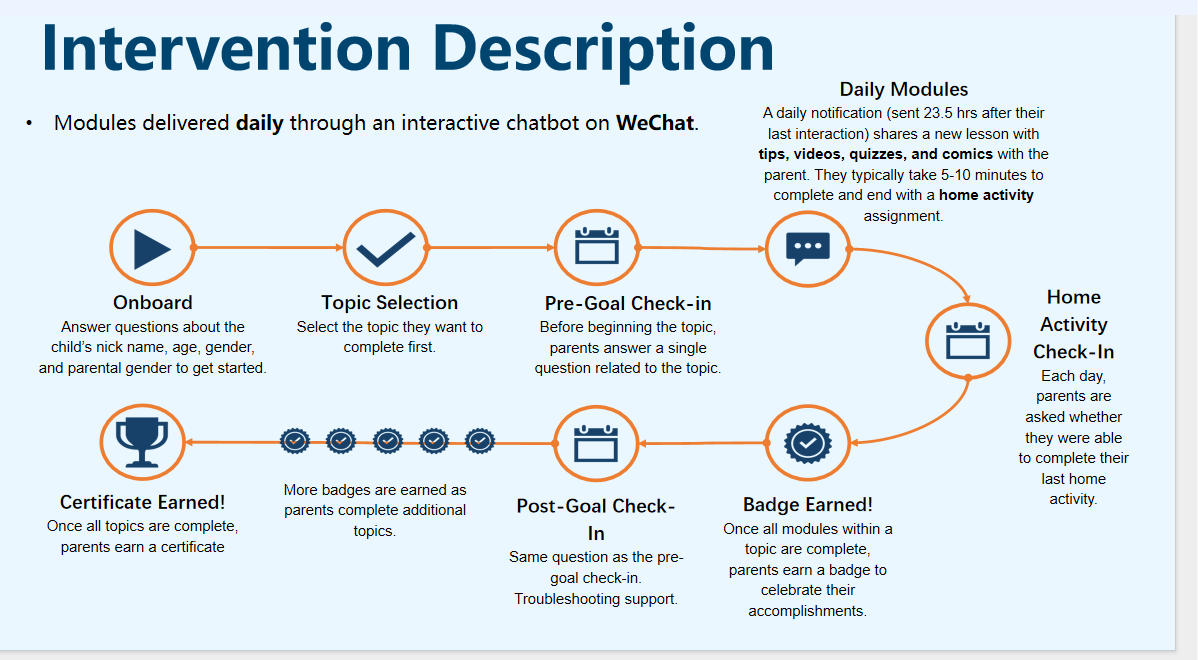


The program was piloted in two settings in China: urban preschool and rural health centers. For the preschool setting, the pilot was conducted in Xinyu, a lower-middle-income city in Jiangxi Province, central China, involving 543 parents. As noted earlier, in addition to chatbot-led delivery, WeChat groups were established for each class. Trained social workers and preschool headteachers facilitated message-based group discussions once or twice per week. These sessions aimed to reinforce key parenting concepts and address parents’ practical concerns. To ensure implementation fidelity, a Facilitator’s Guide was developed, and facilitators were required to complete a fidelity checklist after each session. The program was delivered over a period of 2.5 months.

The other pilot was implemented in two low economic level townships in Gansu Province, located in northwest China, through county-level health cares, township-level Health cares, and village-level public health system. To complement the chatbot-based delivery, trained village doctors conducted biweekly home visits, with each family receiving a total of five visits. These visits followed a structured handbook to ensure standardized delivery and focused on one specific topic per session. The purpose of the home visits was to demonstrate parenting techniques introduced in the digital modules, support parents in practicing the acquired skills, and provide individualized parenting guidance. Village doctors were selected as implementers due to their established trust with local families and their familiarity with community dynamics and family situations (see figure S2).

Figure S2. “Keyushiguang” program workflow


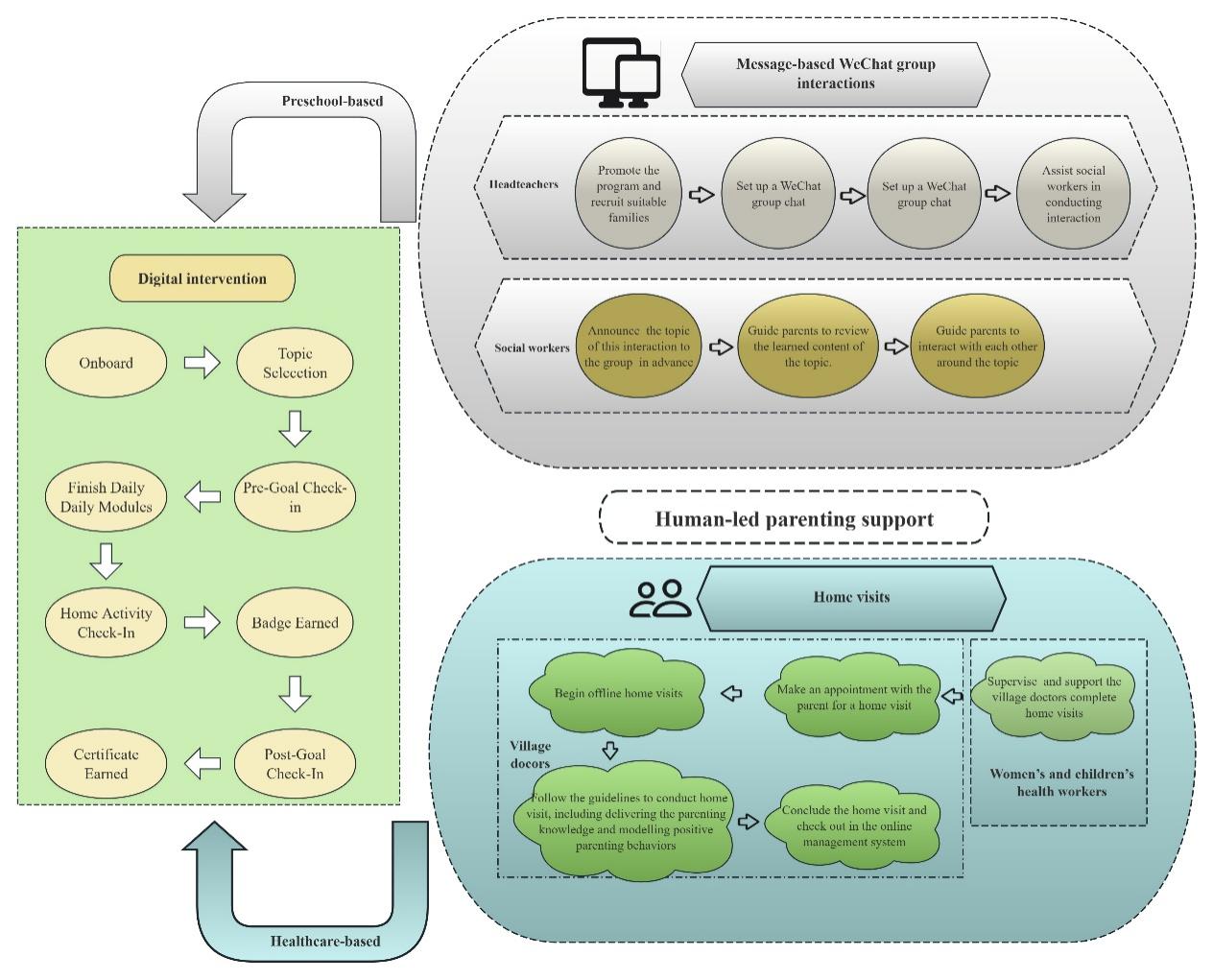

Supplement: Multimedia Appendix 1 [file jmir-v28-e79848-s001.docx]
