# Supplementary material for: Institutionalizing Digital Parenting Programs in Low Resource Settings in China: Comparative Case Study of Health Care and Education Sectors Using the RE-AIM Framework
Source: J Med Internet Res. 2026 Jan 6;28:e79848. doi: 10.2196/79848 (PMC12772938; doi:10.2196/79848)
Supplement: Multimedia Appendix 2 [file jmir-v28-e79848-s002.docx]

# Comparison of two programs

| **Setting** | **Setting Features** | **Stakeholders** | **Eligibility Criteria** | **Digital Intervention** | **Human-led parenting support** |
| --- | --- | --- | --- | --- | --- |
| Urban Preschool | 1. In urban areas of Central China  2. The level of the economy is classified as lower-middle in China  3. Implemented by a local preschool | 1. Preschool leaderships  2. Preschool manager  3. Headteachers  4. Social workers  5.Participants: Caregivers of children aged 36 to 72 months | 1. Caregiver aged 18 and above  2. Not grandparents  3. Primary caregiver of children  4. Own and be able to use smartphones and possess adequate digital literacy | 1. Register in the WeChat account of “Keyushiguang”  2. Select from the 8 parenting topics as the caregivers prefer  3. Learn modules within the topic  4. The system will automatically deliver each module on a daily basis  5. Complete all the modules within that topic  6. Select the next topic  7. Complete all topics  8. Earn a personalized certificate | **Message-based WeChat group interactions**  **1. Preschool headteachers:** Introduce the program, recruit parents, encourage parents to complete online learning, establish WeChat groups, and support online group interactions  **2. Social Workers:** lead group interaction, guiding parents to share parenting experiences, and reinforce course content. |
| Rural Health Centers | 1. In rural areas of Northwest China  2. The level of the economy is classified as low in China  3. Implemented in two township-level health centers | 1. Program officer  2. Government officer in the county and township level healthcare setting  3. Local program coordinator  4. Women and children’s health worker  5. Village doctors  6. Participants: caregivers of children aged 24 to 59 months |  |  | **1. Village doctors:** recruit parents and conduct home visits, encourage parents to complete the daily modules, answer questions, reinforce key parenting knowledge and model parent-child interactions. **2. Local program managers and women’s and children’s health workers:** Supervise and support village doctors |
