# Supplementary material for: Institutionalizing Digital Parenting Programs in Low Resource Settings in China: Comparative Case Study of Health Care and Education Sectors Using the RE-AIM Framework
Source: J Med Internet Res. 2026 Jan 6;28:e79848. doi: 10.2196/79848 (PMC12772938; doi:10.2196/79848)
Supplement: Multimedia Appendix 3 [file jmir-v28-e79848-s003.docx]

# Description of Interview Participants

The characteristics of participants are shown in Table. The study included a total of 83 participants. We conducted one-on-one interviews with 11 key organizational-level stakeholders from both settings, including one program donor, seven local leadership, and four local program managers.

When collecting data from program implementers and participants, we also considered individuals with varying levels of engagement. In the educational setting, we held one FGD with 5 headteachers with higher engagement, one with 4 headteachers with low engagement, and another two FGDs with 7 social workers with similar levels of engagement. Additionally, we conducted individual in-depth interviews with 21 caregivers with high levels of engagement within the chatbot and 5 caregivers with low engagement levels. In the healthcare setting, we organized two FGDs with 18 village doctors who completed the program and one FGDs with two village doctors who declined to participate. We also interviewed 10 parents from rural area, all of whom had completed the online course.

*Table Characteristics of participants*

| **Category** | **The specific role of participants** | **Number** | **Collection method** |
| --- | --- | --- | --- |
| Program donor | Program officer in the healthcare setting | 1 (1 female) | SI |
| Local leadership | County-level healthcare leaders | 2 (2 male) | SI |
|  | Township-level healthcare leaders | 2 (2 male) | SI |
|  | Preschool leaders | 3 (3 female) | SI |
| Local program managers | Program coordinator | 1 (1 female) | SI |
|  | Women’s and Children’s Health Workers | 2 (2 female) | SI |
| Program implementers | Village doctors involved in the program | 18 (5 female) | FGD |
|  | The village doctor who withdrew from the program | 2 (2 male) | FGD |
|  | Social workers in educational setting program | 7 (7 female) | FGD |
|  | Preschool headteachers | 9 (9 female) | FGD |
| Program participants | High Engagement parents | 31 (2 male,10 rural) | SI |
|  | Low Engagement parents | 5 (5 female) | SI |

FG：Focus group discussion; SI: Semi-structured interviews
