# Supplementary material for: Institutionalizing Digital Parenting Programs in Low Resource Settings in China: Comparative Case Study of Health Care and Education Sectors Using the RE-AIM Framework
Source: J Med Internet Res. 2026 Jan 6;28:e79848. doi: 10.2196/79848 (PMC12772938; doi:10.2196/79848)
Supplement: Multimedia Appendix 4 [file jmir-v28-e79848-s004.docx]

# Summary of barriers and facilitators to reach

| **Theme** | **Subtheme** | **Level of influence** | **Setting type** | **Example from qualitative data** |
| --- | --- | --- | --- | --- |
| Facilitators to reach | Program’s strong emphasis on child development | Individual | Both | Parents feel that they should learn some parenting knowledge particularly in areas such as language acquisition, cognitive skills, and socio-emotional growth. |
|  | Strong relationships between program implementers and parents | Individual | Both | Parents think the village doctor and preschool headteacher are credible and have good relationship with them, so they tend to participate in the program delivered by them. |
|  | The perceived authority of the program developers | Individual | Both | Parents believe that the program, designed by researchers from a prestigious university, can be trusted and will benefit them. |
|  | The convenience of learning through a chatbot | Individual | Both | Online learning can take place anytime, anywhere they want. |
|  | Recognition of the value of the program | Individual | Preschool | Some parents in the urban areas reported that the parenting concepts and attitudes conveyed through the program resonated with their own parenting beliefs. |
|  | The perceived professional authority of the preschool teachers | Individual | Preschool-based | Parents consider that preschool teachers are professional in educating children. |
| Barriers to reach | Parenting conservatism | Individual | Both | Some parents are satisfied with their current situation and think there is no need to learn parenting skills. |
|  | Limited time for learning | Individual | Both | Because some parents don’t fully understand the features of the program, they feel that the learning process is time-consuming |
|  | The perceived professional authority of the village doctors | Individual | health center-based | Because village doctors delivered the parenting program for the first time, parents were skeptical about their true intentions behind offering such services. |
