# Supplementary material for: Institutionalizing Digital Parenting Programs in Low Resource Settings in China: Comparative Case Study of Health Care and Education Sectors Using the RE-AIM Framework
Source: J Med Internet Res. 2026 Jan 6;28:e79848. doi: 10.2196/79848 (PMC12772938; doi:10.2196/79848)
Supplement: Multimedia Appendix 5 [file jmir-v28-e79848-s005.docx]

# Summary of barriers and facilitators to adoption

| **Theme** | **Subtheme** | **Level of influence** | **Setting type** | **Example from qualitative data** |
| --- | --- | --- | --- | --- |
| Facilitators to Adoption | Trust in the program developer | Setting | Both | Organizational managers trust in the program developer |
|  | The alignment between the program and their organization’s core functions | Setting | Both | Leaders think organization possessed the capacity and resources to support implementing the program and the program’s initiative aligned with their institutional mandate. |
|  | Organizational empowerment | Setting | Both | Organizational leaders believe that participating in the program can improve the quality of organizational work, the ability of organizational staff, and organizational capacity. |
|  | Task-driven motivation | Individual | Both | Because of the attention of organizational leadership, staff consider this an important task. |
|  | Intrinsic motivation | Individual | Both | The implementers realized that the significance of this pilot and the potential benefits this program could bring to caregivers |
|  | Material incentives | Individual | Health center-based | Appropriate salaries facilitate the participation of village doctors in the program. |
|  | The relevance of the task to the teachers’ professional roles | Individual | Preschool-based | Teachers reported that parenting support was closely tied to their core duties, which can bring benefit to them. |
|  | Prior successful collaboration experiences | Setting | Health center-based | There was a basis for cooperation on other programs before |
| Barriers to adoption | Perceived difficulty of implementation due to limited time | Individual | Both | Implementers are worry about that they do not have enough time to complete the program’s task. |
|  | The irrelevance of the task to the village doctors’ professional roles | Individual | Health center-based | Some village doctors and maternal and child health workers felt their routine work was less directly related to parenting. |
|  | Difficulty in identifying parents | Individual | Health center-based | Some village doctors meet difficulty in identifying parents arose due to factors such as the prevalence of grandparent caregiving in many households. Additionally, geographical barriers and long travel distances to reach rural families further complicated efforts to identify and contact parents for program participation. |
