# Supplementary material for: Institutionalizing Digital Parenting Programs in Low Resource Settings in China: Comparative Case Study of Health Care and Education Sectors Using the RE-AIM Framework
Source: J Med Internet Res. 2026 Jan 6;28:e79848. doi: 10.2196/79848 (PMC12772938; doi:10.2196/79848)
Supplement: Multimedia Appendix 6 [file jmir-v28-e79848-s006.docx]

# Summary of barriers and facilitators to implementation of digital parenting program

| **Theme** | **Subtheme** | **Level of influence** | **Setting type** | **Example from qualitative data** |
| --- | --- | --- | --- | --- |
| Facilitators to implementation | Adequate onboarding training | Setting | Both | The training should allow the implementers to fully understand the program’s content and communicate with them to understand their demands. |
|  | A supportive management system | Setting | Both | A supportive management system includes appropriate incentives and efficient, humanized supervision. |
|  | Timely external support | Setting | Both | Timely, effective, and adequate support from program developers and organizations helped alleviate implementers’ stress. |
|  | The clear work guideline | Individual | Both | To implementers, well-defined procedures contributed to smoother implementation by clearly outlining what needed to be done, when, and how. |
|  | Timing of implementation | Setting | Health center-based | Implementing in off-season for farming make village doctors and caregivers have more spare time. |
| Barriers to implementation | The lack of sense of purpose | Individual | Both | Implementers do not feel valued and think they are just completing tasks. |
|  | Psychological pressures | Individual | Both | Implementers are concerned about the quality of their work. |
|  | Lack of flexibility in work | Setting | Preschool-based | Since the preschool’s work content is more related to parenting, the implementers hope to have more room for their operations. |
|  | The distance is far. | Individual | Health center-based | In the rural areas of Northwest China, the distance between households is far away, affecting village doctors’ home visits. |
