# Supplementary material for: Institutionalizing Digital Parenting Programs in Low Resource Settings in China: Comparative Case Study of Health Care and Education Sectors Using the RE-AIM Framework
Source: J Med Internet Res. 2026 Jan 6;28:e79848. doi: 10.2196/79848 (PMC12772938; doi:10.2196/79848)
Supplement: Multimedia Appendix 7 [file jmir-v28-e79848-s007.docx]

# Summary of barriers and facilitators to maintenance at the setting level of digital parenting program

| **Theme** | **Subtheme** | **Level of influence** | **Setting type** | **Example from qualitative data** |
| --- | --- | --- | --- | --- |
| Facilitators to maintenance at the setting level | The alignment of program content with organizational functions | Setting | Both | The organization’s established functions match the main content of the program. |
|  | The availability of sufficient and appropriate internal human resources | Setting | Both | A team within the organization is capable and stable enough to carry out the program’s tasks. |
|  | The low cost associated with digital delivery | Setting | Both | Digital approach not only helped maintain content quality but also significantly reduced the workload and operational costs for implementing organizations, |
|  | The close relationship between village doctors and local families | Setting | Health center-based | Home visits need a sable, close relationship between delivers and families, village doctors own this strength. |
|  | Make adaptation focus on fitting the organization’s daily work | Setting | Preschool-based | In preschool, program need make adaption in organizational alignment, ensuring the program fit seamlessly within existing school routines |
|  | Make adaptation focus on tailoring the program to specific needs of target populations | Setting | Health center-based | The program needed to be adjusted to include special groups, such as left-behind and migrant children, who make up the majority in the local area and also need this service |
| Barriers to maintenance at the setting level | Institutional dependence on higher government authorization | Setting | Both | Organization must to obtain higher government’s agreement to add this program as their daily work, but it is difficult. |
|  | Challenges in sustaining staff motivation | Setting | Both | How to achieve costly, efficient and motivation implementers once the program became part of daily work. |
|  | Difficulties in generating parental demand for parenting support | Setting | Both | Many parents did not recognize the value or necessity of evidence-based parenting support. |
|  | The cost of village doctors’ salary | Setting | Health center-based | Material incentives were considered more effective to village doctors, as they better compensated for the extra effort required for sustained engagement |
