# Supplementary material for: Institutionalizing Digital Parenting Programs in Low Resource Settings in China: Comparative Case Study of Health Care and Education Sectors Using the RE-AIM Framework
Source: J Med Internet Res. 2026 Jan 6;28:e79848. doi: 10.2196/79848 (PMC12772938; doi:10.2196/79848)
Supplement: Checklist 1 [file jmir-v28-e79848-s008.docx]

# Checklist of iCHECK-DH guidelines.

# iCHECK-DH: Guidelines and Checklist for the Reporting on Digital Health Implementations.

| Section |  | Item | Description | Notes |
| --- | --- | --- | --- | --- |
| Title | 1 | Title(M) | Identification as an implementation report, and description of the implementation in the title and/or keywords | Page 1 - Title |
| Abstract | 2 | Abstract(M) | Provide a summary of the key elements of the implementation report, including a description of the implementation strategy, the intervention, defining the key elements of the implementation and health outcomes and specify the key KPIs/Outputs. We recommend describing the main aspects of the research in the following order: Background - Objectives - Methods - Implementation (Results) - Conclusions - (Optional: Trial Registration). | Page 2-3 - AbstractImplementation outcomes include facilitators and barriers of reach, adoption, implementation and maintenance. |
| Introduction | 3 | Context(M) | Describe the geographical areas, organizations, target populations and implementation context. Consider social, cultural, economic, political, health care and organizational barriers, infrastructures and facilitators that may influence implementation elsewhere. Explicitly highlight whether a national digital health strategy exists and whether implementation is aligned with the strategy.Describe the stage of the implementation (Developing or Adapting Solution / Piloting and Evidence generation / Package and Advocacy /Acceleration / Deploying / Scaling up / Hand over or Complete). | Page 4-6 - BackgroundThe stage of the implementation is in the section of “Cases selection” and Appendix 2. |
|  | 4 | Problem statement(M) | Description of the health care or public health problem, challenge, or deficiency that the implementation aims to address. (If applicable, include a reference to the 'health system challenge' of the WHO Classification of Digital Health Interventions in the description) | Page 5 |
|  | 5 | Similar Interventions(M) | Mention whether this implementation was inspired by another existing one, and if so, what is the added value of your intervention, if any, compared to the initial one? And what, if anything, has been done differently? | It is in the section of “Cases selection” and Appendix 2. This program is based on Parenting for Lifelong Health (PLH). |
| Methods | 6 | Aims and Objectives(M) | Describe the main objectives and the overall aim of the implementation. Describe how these will be measured using predefined primary and secondary outcome(s) and key performance indicators for this implementation and the expected intervention(s).*For example: indicators or proxy-indicators measuring direct health outcomes (e.g., HbA1c for diabetic patients); Key Performance Indicators (e.g., number of users, number of users that are properly trained, user satisfaction); Indicator assessing a particular process (e.g., administrative time for patient admission);* (If there was no evaluation, provide detailed explanation for reasoning) | Page 6-10 - Theoretical underpinnings: RE-AIMWe measured the implementation outcome. The purpose of our intervention is to promote early childhood development. Since this article focuses on the implementation process, the specific outcomes in effectiveness will be reported in other articles. |
|  | 7 | Blueprint summary(M) | Describe the design and key features of the intervention and key points of the implementation strategy and roadmap. | Page 7-9 - Cases selectionMore details can be seen in Appendix 2 (e.g., roadmap ). |
|  | 8 | Technical Design(M) | Reasons for developing or choosing this tool. Does it combine several tools? Provide a brief description of the tool(s) (functionality and architecture) and how it fits into the health enterprise architecture and investment roadmap (if applicable). Indicate whether the solution is based on an existing solution or has been developed or purchased specifically for this intervention.Describe the type of technology used (e.g., AI applications), license of the technology (open source, free, commercial, IP ownership etc.), include code documentation (if available), link to the application, link to wiki or project website. | Page 4-6 - Background Paragraph 4-5 Page 7 -9 - Cases selection |
|  | 9 | Target(M) | The target refers to the focus or recipient of the intervention. It is the specific person, group, system, or problem that the intervention aims to change or improve. The characteristics of the targeted "site(s)" (locations, staff, resources, etc.) for implementation and any eligibility criteria. The population targeted by the intervention and any eligibility criteria. | Page 7-9 - Case selection |
|  | 10 | Data(M) | Describe the data governance, including life cycle (collection, processing, storage, modification, sharing, suppression), the data ownership (mention whether patients actually have access to the data), data protection measures, confidential use of routine data, expected level of data integration, data for research, cross-border data agreement, if any, the applicable legal framework, and how the project complies with it. Data consent: Has patient consent been obtained? Describe the approach to data protection and cybersecurity (e.g. security by design, privacy by design, etc.) and where the data is hosted. (e.g., in-country, cloud based, hybrid model etc.). Describe, if applicable, the government preferences in terms of data policies. | Data governance for the program follows strict privacy regulations. Data collection includes user engagement data (e.g., module completion, participation), and parenting behavior assessments. All data is stored securely with limited access to program staff. Data ownership remains with participants and is accessible to them upon request. Consent has been obtained from all participants. We have obtained the “informed consent” from each participant and make sure the security of data. |
|  | 11 | Interoperability(M) | Describe the interfaces (what other systems does the tool connect to) and the standards that were used (which specific ones and rationale of choice) (e.g., semantic ontologies such ICD as SNOMED, LOINC or technical standards such as HL7 FHIR, etc.). | Because this is a parenting program, it is temporarily not related to interfaces and standards, but it operates within the WeChat ecosystem, which is widely used for communication and information delivery in China. |
|  | 12 | Participating entities(M) | Describe the implementing organization(s): Type of organisation(s), mission, leadership, vision, etc.Government involvement: Describe whether the government was involved in the implementation, at what level and at what stage(s).Partners: Describe all partners (organisations) and their role in the implementation.Funders: List all actors and stakeholders who have funded or invested in the development of the implementation (if different from the implementation, e.g. using an existing digital health intervention). Indicate their level of involvement in terms of funding.Mention which entity will own the final product and intellectual property after the implementation phase. | Page 7-9 - Cases selectionMore details can be seen in Multimedia appendix 3. |
|  | 13 | Budget Planning(M) | Describe the planned budget for implementation (include costs such as change management, user training, project management, technology pricing, total cost of ownership). If possible, include actual costs, otherwise describe the range or percentage of the total budget. Indicate the period covered by the budget. Describe the budget for the intervention (e.g. development, purchase or adaptation of a free tool); if possible include real costs, otherwise describe as a percentage of the total budget. Indicate the duration covered by the budget. | NM |
|  | 14 | Sustainability(M) | Describe the Business model including the sustainability model (financial, economic, environment etc.). If possible, put outcomes in relation to cost to assess sustainability. Describe long term exit strategies, and all dimensions considered to sustain the project after the end of funding. If applicable, describe potential institutionalization of the project. | The sustainability of the program relies on integrating it with local education and healthcare systems. The focus is on exploring pathways for institutionalization within these systems, ensuring the program can be sustained over time. Its digital nature enables low-cost, scalable delivery, and with successful implementation, there is potential for long-term sustainability in both urban and rural settings. |
| RESULTS | 15 | Coverage(M) | Describe whether the coverage of implementation is international, national, regional or at the level of e.g. municipalities. If coverage is sub-national, describe the regions. Provide information on the relative importance of the coverage (e.g. % of eligible population covered). | Page 15- ResultThe paragraph 1 in the section of “Reach-facilitators and barriers”. |
|  | 16 | Outcomes(M) | Primary and other outcome(s) of the implementation. Detail the actual outcomes, using the pre-defined outcome measures (if applicable). | Page 15 -35 - ResultThe paragraph 1 in the section of “Reach-facilitators and barriers”.The paragraph 1 in the section of “Adoption-facilitators and barriers”.The paragraph 2 in the section of “Implementation-facilitators and barriers”. |
|  |  | Lessons learned(M) | Describe any lessons learned from the implementation experience that could be used to improve future outcomes. This could include, but is not limited to, success factors, implementation challenges or budget considerations.Success factors: Describe factors that positively influenced the implementation (e.g. involvement of key stakeholders). Also describe contextual factors that may have positively influenced the results (e.g. new legal requirements that facilitated adoption).Challenges to implementation: Describe challenges (process-related, such as resistance to change, but also technical). Include contextual factors that may have affected the achievement of outcomes such as an unexpected change of government, or 'opposing key players' who, despite potential participation, may hinder implementation (e.g. software companies managing regional digital health may act as barriers to innovation).Budget: Describe whether the implementation budget was adhered to, and if not, why not. Also detail the expected operational costs (e.g. licence, maintenance, human resources, updates to in-house developments) to estimate the total cost of ownership. Include real costs, otherwise describe them as a percentage of the total budget.What recommendations can be drawn from the lessons learned? |  |
|  | 17 |  |  | Page 15 -35 - ResultSuccess factors are the facilitators of RE-AIM.Challenges to implementation are the facilitators of RE-AIM.Budget not mention in article but it had been adhered. |
|  | 18 | Unintended consequences(NM) | Describe unintended consequences (positive or negative), harms or negative side-effects (if any). | NM |
| Discussion | 19 | Conclusion(M) | Summary of the conclusions and future implications. | Page 34 - Conclusion |
| General | 20 | General(NM) | If applicable, include statement(s) on regulatory approvals (including, as appropriate, ethical approval, governance approval), trial or study registration (availability of protocol), and conflicts of interest. For implementation reports with a research component, ethical approval or a waiver from an appropriate ethics committee is required. For those without a research component, ethical considerations may still be relevant, but do not necessarily require approval or waiver. Authors may consult [this article](https://implementationscience.biomedcentral.com/articles/10.1186/1748-5908-6-32) for further guidance on ethical considerations in their specific context" | Page 12 - Ethical approvalPage 39 - Acknowledgement |
